# Further observations on scorpion genera *Hadrurus* and *Hoffmannihadrurus* (Scorpiones, Caraboctonidae)

**DOI:** 10.3897/zookeys.59.546

**Published:** 2010-10-01

**Authors:** Michael E Soleglad, Victor Fet

**Affiliations:** 1 P.O. Box 250, Borrego Springs, California 92004, USA; 2 Department of Biological Sciences, Marshall University, Huntington, West Virginia 25755-2510, USA

**Keywords:** Caraboctonidae, *Hadrurus*, *Hoffmannihadrurus*, neobothriotaxy, Baja California Sur, Mexico

## Abstract

Multiple populations of Hadrurus pinteri from Baja California Sur, Mexico have been examined. It is demonstrated that the southern populations of this species have a larger number of accessory trichobothria (neobothriotaxy) than the northern populations, numbers exceeding the maximum currently recorded for the genus. Examination of carapace and chela coloration and its patterns show a close affinity between Hadrurus pinteri and the dark phase of Hadrurus concolorous. A new morphometric ratio of the carapace is defined that distinguishes Hadrurus from Hoffmannihadrurus, further supporting the monophyly of the latter genus.

## Introduction

Fet and Soleglad (2008) presented a cladistic analysis of scorpion superfamily Iuroidea. In this analysis they demonstrated the monophyly of genus Hoffmannihadrurus, a taxon which had been recently synonymized by Francke and Prendini (2008). In this present contribution, new information is presented from the evaluation of additional specimens of Hadrurus pinteri Stahnke, 1967 and Hadrurus concolorous Stahnke, 1967, all from Baja California Sur, Mexico. This information is relevant to the cladistic analysis of Fet and Soleglad (2008) as follows: 1) it supports their hypothesis that the northern populations of Hadrurus pinteri are losing accessory trichobothria as demonstrated by a larger number being found in the southern populations; 2) analysis of coloration and its patterns of the carapace and chela of Hadrurus pinteri show close affinities to the dark phase of Hadrurus concolorous, not Hoffmannihadrurus gertschi, as proposed by Francke and Prendini (2008); and 3) a new morphometric ratio involving the carapace is defined which can be used to diagnostically separate genera Hadrurus and Hoffmannihadrurus. Items 2 and 3 further support the result of Fet and Soleglad (2008) that confirms the validity of genus Hoffmannihadrurus.

## Material and methods

### Abbreviations

ABDSP, Anza-Borrego Desert State Park, San Diego and Riverside Counties, California, USA.

### Depositories

CAS, California Academy of Sciences, San Francisco, California, USA;MES, Personal collection of Michael E. Soleglad, Borrego Springs, California, USA;VF, Personal collection of Victor Fet, Huntington, West Virginia, USA.

### Material

The following material was examined for analysis and/or illustrations provided in this paper. It must also be noted that many observations and statistics provided in this paper are augmented, in part, from other data previously collected and discussed in Soleglad (1976), Fet et al. (2001, 2004), Soleglad and Fet (2004), and Fet and Soleglad (2008).

### Genus Hadrurus Thorell, 1876:

Hadrurus arizonensis arizonensis Ewing, 1928: Carrizo Badlands, Vallecito Creek, ABDSP, California, USA ♂ (MES), 41.2 mi. E San Luis, Sonora, Mexico, ♂ (MES); Hadrurus arizonensis austrinus Williams, 1970: Oakies Landing, Baja California, Mexico, ♂ ♀ (MES); Hadrurus concolorous Stahnke, 1969: 8 km S Tambobiche, Baja California Sur, Mexico, ♀ (CAS), 5 mi SW San Miguel de Comondú, Baja California Sur, Mexico, ♂ (CAS), Santa Rosalia, Baja California Sur, Mexico, ♂ ♀ (MES), Las Bombas, Baja California Sur, Mexico, ♂ (MES); Hadrurus hirsutus (Wood, 1863): Cabo San Lucas, Baja California Sur, Mexico, 2 ♂ (MES); Hadrurus obscurus Williams, 1970: Indian Gorge, ABDSP, California, USA, 2 ♀ (MES); Hadrurus pinteri Stahnke, 1969: Oakies Landing, Baja California, Mexico, 3 ♀ 4 ♂ 3 J (MES), Arroyo Calamajué, Baja California, Mexico, 2 ♀ (MES), Punta Trinidad, Baja California Sur, Mexico, 2 ♂ (CAS), San Ignacio, Baja California Sur, Mexico, 2 ♀ (CAS), Bahia Concepción,Baja California Sur, Mexico, ♀ (CAS), 5 mi SW San Miguel de Comondú, Baja California Sur, Mexico, ♂ (CAS), 5–10 mi SW San Miguel de Comondú, Baja California Sur, Mexico, 5 ♂ 4 ♀ (CAS), 5.6 mi SW Loreto, Baja California Sur, Mexico, ♀ (CAS), 7.2 mi SW Loreto, Baja California Sur, Mexico, ♂ (CAS), 8.3 mi SW Loreto, Baja California Sur, Mexico, ♀ (CAS), 14.7 mi SW Loreto, Baja California Sur, Mexico, ♀ (CAS), Isla Danzante (NW side), Baja California Sur, Mexico, 5 ♀ (CAS), Agua Verde Bay, Baja California Sur, Mexico, 2 ♀ (CAS); Hadrurus spadix Stahnke, 1940: Hawthorne, Mineral Co., Nevada, USA (VF).

### Genus Hoffmannihadrurus Fet & Soleglad, 2004:

Hoffmannihadrurus aztecus (Pocock, 1902): Tehuacán, Puebla, Mexico, 3 ♂ 1 ♀ (MES), Tomellín, Oaxaca, Mexico, ♂ (MES); Hoffmannihadrurus gertschi (Soleglad, 1976), Azcala, Guerrero, Mexico, paratype ♀ (CAS), Iguala, Guererro, Mexico, ♀ (MES).

## Neobothriotaxy in Hadrurus pinteri

Neobothriotaxy in Hadrurus was first reported by Gertsch and Soleglad (1972: figs 96–107) when the first trichobothrial pattern for this genus was illustrated for Hadrurus arizonensis. The unusual and complicated pattern exhibited in this genus was later investigated by Soleglad (1976) where he illustrated and provided trichobothria statistics of the chela for eight species (now divided into two genera, Hadrurus and Hoffmannihadrurus). This analysis of Soleglad (1976) involved the study of over 200 specimens and the description of new species Hoffmannihadrurus gertschi (assigned to Hadrurus at the time). The most important result of this study was that the major species groups could be diagnosed using chelal trichobothrial patterns alone. These diagnoses were based in most part on the presence/absence and numbers of accessory trichobothria on three surfaces of the chela, ventral, internal, and external. In other words, neobothriotaxy could be used in large part to distinguish the species. Years later, Fet et al. (2001) provided the first systematic analysis of the six species of Hadrurus using DNA. Supporting this analysis was morphology analyzed from a cladistic perspective; again, neobothriotaxy contributed several characters. The original data set of Soleglad (1976) was expanded by over 50 % with many additional specimens being added, primarily in the “*hirsutus*” group. At this time the sample set was over 600 (i.e., both chelae are considered). Based on the large collection of neobothriotaxy data from these specimens as well as from chactoid scorpion genera (Anuroctonus and Euscorpius), data that were closely correlated with the specimen’s geographical locality, Soleglad and Fet (2004: 102–106) presented an “accessory trichobothria loss” hypothesis. We will not present this hypothesis here, it is discussed in detail in the aforementioned reference as well as in the most recent work of Fet and Soleglad (2008: 273–277). In the latter reference, germane to this study, it was suggested that the accessory trichobothria were being lost in the northern populations of Hadrurus pinteri, presumably the direction of the radiation of this species. I.e., the assumption being the species origin is in Baja California Sur, the primary location of the “*hirsutus*” group, which also includes species Hadrurus concolorous and Hadrurus hirsutus as well as Hadrurus pinteri. However, Fet and Soleglad (2008) had very little Hadrurus pinteri material from the southern area of Baja California, so this assumption of accessory trichobothria loss in the northern half was not based on substantial data.

**Figure 1.**
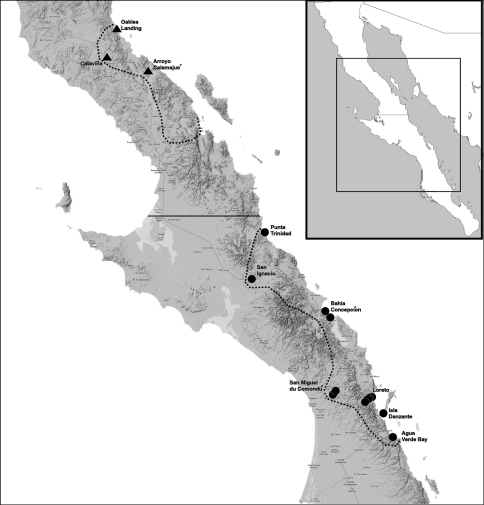
Reported distribution of Hadrurus pinteri. The two dotted line areas partition Hadrurus pinteri distribution into the northern and southern portions of Baja California Peninsula, Mexico. These areas, in general, denote the original distribution reported by Williams (1970: fig. 45). Solid icons indicate localities of specimens used in our statistics, triangles northern specimens and circles southern specimens. Note that localities indicated with icons are labeled with larger text. Boxed inset map indicates the portion of Baja California, Mexico shown in foreground terrain map.

In this study, we have analyzed a collection of southern populations of Hadrurus pinteri from the California Academy of Sciences. We tabulated the number of chelal accessory trichobothria of the ventral, internal, and external surfaces and compared it to the data of the northern populations. Figure 1 shows the geographic localities of Hadrurus pinteri from which the accessory trichobothria data are derived. The northern samples, totaling 13 specimens, are from the northern half of their known range, the majority of specimens from Oakies Landing. The most southern of these specimens is from the Arroyo Calamajué. The southern samples, comprised of 28 specimens, span the entire southern range originally outlined by Williams (1970). The most northern specimens, from Punta Trinidad, are farther north than originally reported by Williams (1970), these specimens not listed in his material examined. San Miguel de Comondú provided the most specimens, ten in number. Interestingly, the range of Hadrurus pinteri, as stated by Williams (1970) and shown in our map (Fig. 1) is disjunct, roughly 150 km separating the northern and southern ranges. Hadrurus pinteri, as reported by Williams (1970: 18): “… was never found in predominantly sandy habitats or away from habitats of volcanic origin …”. As can be seen in Fig. 1, the terrain is certainly mountainous, the eastern portion of the peninsula volcanic from Puertocitos to well south of Loreto. What is interesting in this map is that the area of disjunction between the two ranges of Hadrurus pinteri is also volcanic leading to the conclusion that Hadrurus pinteri distribution is probably not disjunct. The gap in reported localities is probably due to the lack of collecting in this area. Williams and his associates conducted their monumental collecting expeditions in Baja California during the late 1960s, Williams (1980: 1), in his monograph on the scorpions of Baja California, Mexico, reported that 60,000 specimens were examined from Baja California! However, during this time, access to this particular eastern area of Baja California was difficult if not impossible, as the primary road (unpaved during the time) was on the western side of the peninsula.

Table 1 shows the statistical breakdown of accessory trichobothria for the entire “*hirsutus*” group, involving more than 260 samples. Of importance to this discussion is the breakdown of Hadrurus pinteri into its northern and southern populations. As predicted by Fet and Soleglad (2008), we see that the southern populations of Hadrurus pinteri exhibited larger numbers of accessory trichobothria than the northern samples in two of three chelal surfaces, the internal accessory trichobothria showing slightly higher numbers in the northern populations. For the ventral series, the mean value of the southern populations was 8 % larger than that seen in the northern populations, roughly two additional accessory trichobothria on an average. Similarly, the external surface of the southern populations exhibited over a 7 % mean value difference. For the internal series, the northern populations had 1.4 % more accessory trichobothria on an average, though the standard error range maximum value was slightly larger in the southern population.

**Table 1.** Statistics showing neobothriotaxy of the pedipalp chela of the Hadrurus “*hirsutus*” group. In particular, two disjunct populations of Hadrurus pinteri are contrasted showing that for two of the three trichobothrial series, the southern population (i.e., Baja California Sur) has the largest number of accessory trichobothria, roughly a 7.5 % increase. MVD = mean value difference; p-value = Anova output. Statistical data group: minimum–maximum (mean) (±SDEV) [N] {standard error range} (coefficient of variability) (SDEV/mean). * includes orthobothriotaxic trichobothria V1–V4. Many of the statistics are from previous studies, as well as new material examined in this project. See Soleglad (1976), Soleglad and Fet (2004), Fet et al. (2004), and Fet and Soleglad (2008).

Chela Neobothriotaxy Statistics for Hadrurus “*hirsutus*” Group		
**Hadrurus pinteri, Baja California, Mexico (“Norte” half of peninsula)**		
Ventral*	22–26 (24.292) (±1.429) [024] {22.863–25.721} (0.059)	
**Internal**	5–6 ( 5.692) (±0.471) [026] { 5.222–6.163} (0.083)	
**External**	3–4 ( 3.500) (±0.511) [024] { 2.989–4.011} (0.146)	
**Hadrurus pinteri, Baja California Sur, Mexico (“Sur” half of peninsula)**		
Ventral*	23–32 (26.245) (±1.870) [053] {24.375–28.115} (0.071)	MVD/p-value: Sur > 8.0%/2.06E-05
**Internal**	5–7 ( 5.611) (±0.564) [054] { 5.048–6.175} (0.100)	MVD/p-value: Sur < 1.4%/ 0.527192
**External**	3–5 ( 3.759) (±0.612) [054] { 3.147–4.372} (0.163)	MVD/p-value: Sur > 7.4%/ 0.074043
**Hadrurus pinteri, all of Baja California, Mexico**		
Ventral*	22–32 (25.636) (±1.960) [077] {23.677–27.596} (0.076)	
**Internal**	5–7 ( 5.637) (±0.534) [080] { 5.104–6.171} (0.095)	
**External**	3–5 ( 3.679) (±0.592) [078] { 3.087–4.272} (0.161)	
**Hadrurus concolorous**		
Ventral*	15–20 (17.804) (±1.170) [158] {16.634–18.974} (0.066)	
**Internal**	3–5 ( 4.154) (±0.493) [162] { 3.661–4.647} (0.119)	
**External**	1–2 ( 1.226) (±0.420) [159] { 0.807–1.646} (0.342)	
**Hadrurus hirsutus**		
Ventral*	14–16 (15.682) (±0.568) [022] {15.114–16.250} (0.036)	
**Internal**	4–5 ( 4.048) (±0.218) [021] { 3.829–4.266} (0.054)	
**External**	1–2 ( 1.048) (±0.218) [021] { 0.829–1.266} (0.208)	

**Figures 2–4.**
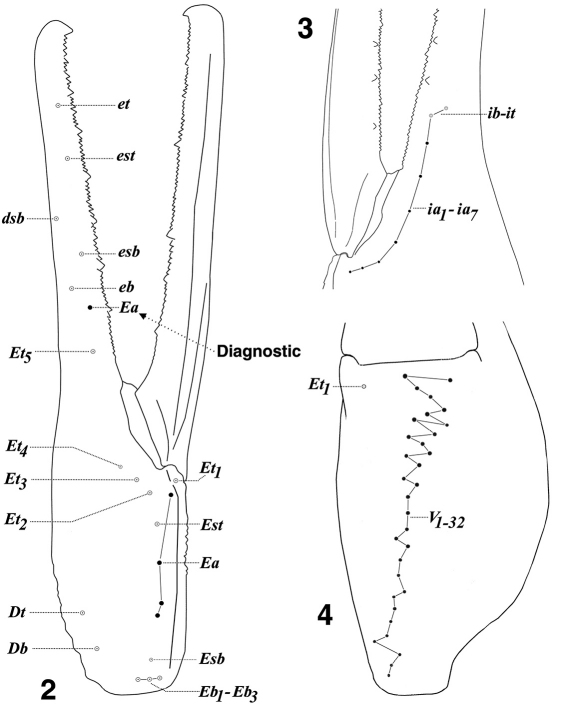
Hadrurus pinteri, chelal neobothriotaxy showing examples of patterns with the *largest number* of accessory trichobothria (closed circles). **2** External view of left chela (reversed), juvenile male, Punta Trinidad, Baja California Sur, Mexico, showing five external accessory (*Ea*) trichobothria, including the diagnostic *Ea* on the base of the fixed finger (*db*, *dst*, and *dt* are not shown) **3** Internal view of left chela (reversed), adult female, Loreto, Baja California Sur, Mexico, showing *nine* internal trichobothria, including *ib–it* and seven accessory trichobothria (*ia*). Note that the accessory trichobothria reduce in size somewhat as they occur basally **4** Ventral view of right chela, adult female, Bahia Concepción, Baja California Sur, Mexico, showing 32 ventral trichobothria, including V^1^–V^4^, and Et^1^ (note that V^1^–V^4^ are not distinguished from accessory trichobothria).

Of particular interest, we found that the southern populations of Hadrurus pinteri exhibited the largest number of accessory trichobothria found in the three individual chelal surfaces for the entire genus Hadrurus (as well as for its sister genus Hoffmannihadrurus). Previously, based on data presented in Fet et al. (2004), the largest number of internal, ventral, and external accessory trichobothria were seven (Hadrurus arizonensis), 27 (23 accessory, Hadrurus pinteri), and four (Hadrurus pinteri and Hoffmannihadrurus gertschi), respectively. In these newly examined populations of Hadrurus pinteri we found seven internal accessory trichobothria in two specimens, from Loreto and Agua Verde Bay, thus matching the maximum counts found in Hadrurus arizonensis. For the latter species, four specimens exhibited seven internal accessory trichobothria, one from ABDSP in California, and three from Sonora, Mexico, all belonging to the pale form of this species (previously referred to as Hadrurus arizonensis pallidus; synonymized by Fet et al., 2001). Now, comparing Hadrurus pinteri to Hadrurus arizonensis, which includes subspecies Hadrurus arizonensus austrinus, we see that the former species mean value is slightly larger, 5.637 vs. 5.490 (note that 253 samples represent Hadrurus arizonensis). For the southern populations of Hadrurus pinteri, we found a specimen with 32 ventral trichobothria (28 accessory), exceeding the previous largest number by five. In addition to the largest ventral number, one specimen (from Punta Trinidad) exhibited 30 ventral trichobothria, five with 29, and four with 28. The largest ventral trichobothria count from the northern half of Baja California is 26. The mean value difference between the two localities in Baja California is 8 %. We encountered no less than five instances of five external accessory trichobothria (Punta Trinidad, San Miguel de Comondú, and Isla Danzante). This count includes the diagnostic and unique accessory trichobothrium found on the base of the fixed finger. Figures 2–4 illustrate examples of these large accessory trichobothria numbers for each chelal surface.

## Coloration and Patterns of Hadrurus and Hoffmannihadrurus

While examining the southern populations of Hadrurus pinteri, we discovered that two of the specimens were in fact not Hadrurus pinteri, but Hadrurus concolorous. Based on coloration and patterns these two specimens certainly looked like Hadrurus pinteri, only after detailed trichobothrial analysis could we isolate the two specimens from Hadrurus pinteri. One specimen, from Tambobiche, had a somewhat small number of accessory trichobothria, only 14–15 ventral and three internal. The other specimen, from San Miguel de Comondú, exhibited 18–19 ventral, five internal, and one external accessory trichobothria. In both specimens, the diagnostic accessory trichobothrium on the fixed finger was absent. Of special interest, the specimen from San Miguel de Comondú was contained in a vial with a large Hadrurus pinteri male, thus they were collected together.

**Figures 5–16.**
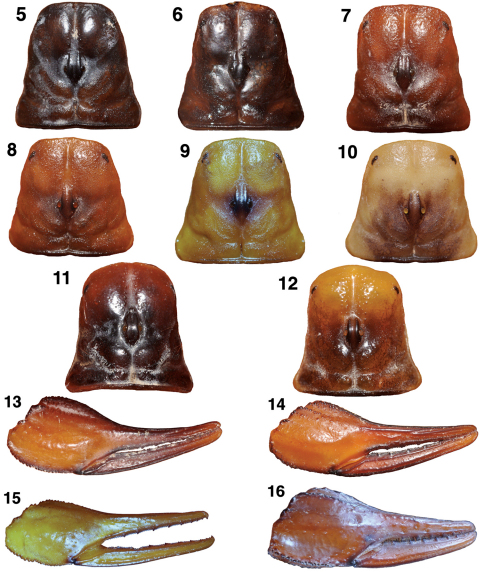
Carapaces and chelae of Hadrurus and Hoffmannihadrurus showing the variability in coloration and its patterns. Of particular interest is the range of variability in Hadrurus concolorous (**Figs 6–9, 14–15**) exhibiting dark coloration patterns essentially identical to Hadrurus pinteri (**Figs 5, 13**) to pale yellow with little or no patterns. Also of interest is the darken posterior half of the carapace in Hoffmannihadrurus gertschi, matching the same area that is also darkened in Hoffmannihadrurus aztecus **5, 13** Hadrurus pinteri, male, San Miguel de Comondú, Baja California Sur, Mexico **6** Hadrurus concolorous, female, Tambobiche, Baja California Sur, Mexico **7, 14** Hadrurus concolorous, male, San Miguel de Comondú, Baja California Sur, Mexico (same locality as Hadrurus pinteri in Fig. 5) **8** Hadrurus concolorous, male, Santa Rosalia, Baja California Sur, Mexico **9, 15** Hadrurus concolorous, male, Las Bombas, Baja California Sur, Mexico 10 Hadrurus hirsutus, male, Cabo San Lucas, Baja California Sur, Mexico **11, 16** Hoffmannihadrurus gertschi, female paratype, Azcala, Guerrero, Mexico **12** Hoffmannihadrurus aztecus, female, Tehuacán, Puebla, Mexico.

Figures 5–9 show the carapaces of Hadrurus pinteri, the two Hadrurus concolorous misidentified for Hadrurus pinteri, and two additional color phases of Hadrurus concolorous. The carapaces of Hadrurus pinteri and the Hadrurus concolorous from Tambobiche are indistinguishable, both uniformly dark in color. The carapace of the specimen from San Miguel de Comondú is lighter in color, more close to the reddish specimen from Santa Rosalia (Fig. 8). The specimen from the sand dune area in Las Bombas (Fig. 9) is typical of Hadrurus concolorous, as indicated by its name “concolorous”.

The chela of Hadrurus pinteri (Fig. 13) and the Hadrurus concolorous from San Miguel de Comondú (Fig. 14) are indistinguishable. Again, the chela from the Las Bombas specimen (Fig. 15) is typical of the “concolorous” phase of Hadrurus concolorous.

Figures 11–12 show the carapacial coloration and patterns of Hoffmannihadrurus gertschi and Hoffmannihadrurus aztecus. Although the carapace of Hoffmannihadrurus gertschi is considerably darker, its interocular area is lighter in color, exhibiting similar light/dark patterns as seen in its sister species Hoffmannihadrurus aztecus. It is clear that the carapace of Hoffmannihadrurus gertschi is not patterned as in Hadrurus pinteri (Fig. 5). Similarly the chela of Hoffmannihadrurus gertschi (Fig. 16) is darker than that seen in Hadrurus pinteri (Fig. 13). Francke and Prendini (2008) attempted to associate the carapace and chelal coloration/patterns of Hoffmannihadrurus gertschi to that of Hadrurus pinteri (their “coloration” characters 2–3, and 5). However, the dark color phase of Hadrurus concolorous is much closer to Hadrurus pinteri, a more reasonable association since the two species are closely related and share the same geographical area! The above observations of essentially identical coloration in Hadrurus pinteri and the dark phase of Hadrurus concolorous in conjunction with the lack of close compliance to Hoffmannihadrurus gertschi further endorses the observations of Fet and Soleglad (2008: 272–273).

## Carapace Morphometrics of Hadrurus and Hoffmannihadrurus

**Figure 17.**
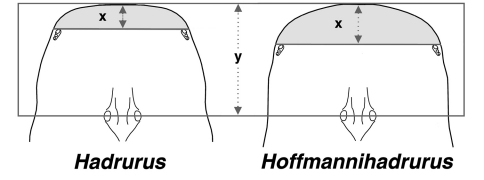
Method of measurement of carapace for genera Hadrurus and Hoffmannihadrurus for determining morphometric *ratio anterior_edge_ length* (x) / *median_tubercle_position* (y). Shaded area indicates *anterior_edge_length*. Diagrammatic drawings based on Hadrurus pinteri and Hoffmannihadrurus gertschi.

While studying the carapacial coloration and its patterns in Hadrurus and Hoffmannihadrurus we observed that the convexed anterior edge exhibited in both genera was considerably more exaggerated in Hoffmannihadrurus. This is quite visible in the photographs presented in Figs 5–12 as well as in Fet et al. (2004: figs 57–58) where Hadrurus pinteri and Hoffmannihadrurus aztecus are shown. We analyzed this difference in the anterior edge of the carapace in both genera, including all species and subspecies of Hadrurus. Several morphometrics were obtained in an attempt to quantify this visible difference between the two genera. From these morphometrics we discovered that the anterior edge (measured from the lateral eyes to the distal aspect of the carapace, see Figure 17) was longer in Hoffmannihadrurus. We concluded that the more overt convexed aspect seen in the Hoffmannihadrurus carapace contributes directly to its elongation. This was evident from two ratios we constructed: the anterior edge length as compared to the carapace length and the anterior edge length as compared to the position of the median eyes. The results derived from these two ratios are nearly identical, so we present here only the latter of the two ratios.

Figure 17 illustrates exactly how these two measurements are taken and Table 2 presents the results involving 30 samples spanning all species of Hadrurus (20 samples), and Hoffmannihadrurus (ten samples). The sampling included four Hoffmannihadrurus gertschi, six Hoffmannihadrurus aztecus, and ten samples each from the Hadrurus “arizonensis” group (i.e., both Hadrurus arizonensis subspecies, Hadrurus obscurus, and Hadrurus spadix), and the “*hirsutus*” group (i.e., Hadrurus pinteri, Hadrurus concolorous, and Hadrurus hirsutus). The mean value differences of this morphometric ratio between these genera is 47.7%, implying that in Hoffmannihadrurus, the anterior edge of the carapace is roughly 50 % longer than in Hadrurus. [note that the carapace of Hadrurus concolorous from Tambobiche (Fig. 6) was not included in the morphometric sampling due to its obvious damaged anterior edge.] Other relevant statistical indicators are no overlap of the absolute range, over 200 % separation of the standard error range, and a very small anova p-value of 6.12E-16.

**Table 2.** Statistics showing differences in the length of the carapace anterior edge in subfamily Hadrurinae based on the following morphometric ratio: *anterior_edge_length* / *median_tubercle_position*. See Fig. 17 for methods of measurement. Data shows that the anterior edge of Hoffmannihadrurus is approximately 48 % longer than in Hadrurus. Large standard error range separation and a very small *p-value* from variance analysis further support the significant statistical difference between the two genera. Statistical data group: minimum–maximum (mean) (±SDEV) [N] {standard error range} (coefficent of variability). * Mean value difference, standard error range separation, and analysis of variance. Statistical data derived from specimens examined and the following references: Williams (1970), Stahnke (1971), Soleglad (1976), and Fet et al. (2004).

Carapace Anterior Edge Ratio for Subfamily Hadrurinae	
**Hoffmannihadrurus**	
**Hoffmannihadrurus gertschi**	0.351–0.383 (0.3663) [4]
**Hoffmannihadrurus aztecus**	0.327–0.368 (0.3461) [6]
**Genus**	0.327–0.383 (0.3542) (±0.0166) [10] {0.338–0.371} (0.047)
**Hadrurus**	
**Hadrurus pinteri**	0.219–0.257 (0.2320) [4]
**Hadrurus concolorous**	0.219–0.246 (0.2331) [4]
**Hadrurus hirsutus**	0.220–0.250 (0.2349) [2]
**Hadrurus arizonensis**	0.233–0.267 (0.2553) [4]
**Hadrurus obscurus**	0.206–0.253 (0.2322) [4]
**Hadrurus spadix**	0.256–0.260 (0.2584) [2]
**Genus**	0.206–0.267 (0.2399) (±0.0185) [20] {0.218–0.262} (0.092)

Statistical comparisons between genera: MVD * = 47.7 SERS * = 238.8 ANOVA *p–value = 6.12E-16

Fet and Soleglad (2008) presented a detailed cladistic analysis of the superfamily Iuroidea demonstrating monophyly of the families Iuridae and Caraboctonidae, the subfamilies Caraboctoninae and Hadrurinae, and the genera Hadrurus and Hoffmannihadrurus. This resulted in the reestablishment of the genus Hoffmannihadrurus, which had been recently synonymized by Francke and Prendini (2008). Fet and Soleglad’s (2008) approach was to present their cladistic analysis in three successive layers: fundamental characters, low-level characters, and characters based on the accessory trichobothria loss hypothesis. The first layer presented characters that dealt with higher-level systematic aggregates, characters that were assumption- and hypothesis-free. Low-level characters dealt with coloration and its patterns, setation, and etc., which are generally species-level characters. The last layer of characters, based on the loss hypothesis, as its name implies, is a hypothesis, thus formed from a set of assumptions. Although the hypothesis has been studied across three separate scorpion groups involving thousands of scorpions, it is still a hypothesis. It must be noted here that the monophyly of Hoffmannihadrurus was demonstrated at the first layer of cladistic analysis, using only fundamental characters. As the other two layers were added, successively, this monophyly was further demonstrated with larger support (i.e., more characters and greater bootstrap/jackknife support).

The new character described above showing differences in the anterior edge length between Hadrurus and Hoffmannihadrurus represents the fifth new character supporting monophyly of Hoffmannihadrurus (i.e., four were previously identified by Fet and Soleglad (2008)); these five characters were not included in the analysis by Francke and Prendini (2008). In this study, we added this character to the original cladistic analysis presented by Fet and Soleglad (2008: 265) by adding a new state (= 3) to the fundamental character 23 (carapace anterior edge). We then reinitiated the fundamental and final character cladistic sequences with the following results: for the fundamental sequence, instead of three MPT’s (most parsimonious trees) we obtained two; the bootstrap/jackknife results for monophyly of Hoffmannihadrurus improved from 68/66 % to 88/83 % (that is, 88/83 percent of the 5000 pseudoreplicates supported this monophyly); for the final sequence, bootstrap/jackknife results for monophyly improved from 99/97 % to 100/98 %; and finally, character 23: state = 3 distributed unambiguously on the Hoffmannihadrurus node in both sequences, which is clearly a demonstrated synapomorphy for genus Hoffmannihadrurus. Refer to Fet and Soleglad (2008) for details in this cladistic analysis and definitions of specialized terminology.
